# Mixed strongyle parasite infections vary across host age and space in a population of feral horses

**DOI:** 10.1017/S0031182024001185

**Published:** 2024-10

**Authors:** Sangwook Ahn, Elizabeth M. Redman, Stefan Gavriliuc, Jennifer Bellaw, John S. Gilleard, Philip D. McLoughlin, Jocelyn Poissant

**Affiliations:** 1 Faculty of Veterinary Medicine, University of Calgary, Calgary, AB, Canada; 2 M.H. Gluck Equine Research Center, Department of Veterinary Science, University of Kentucky, Lexington, KY, USA; 3 Department of Biology, University of Saskatchewan, Saskatoon, SK, Canada

**Keywords:** disease ecology, DNA metabarcoding, gastrointestinal parasite, ITS2, mixed infection, nemabiome

## Abstract

Identifying factors that drive among-individual variation in mixed parasitic infections is fundamental to understanding the ecology and evolution of host–parasite interactions. However, a lack of non-invasive diagnostic tools to quantify mixed infections has restricted their investigation for host populations in the wild. This study applied DNA metabarcoding on parasite larvae cultured from faecal samples to characterize mixed strongyle infections of 320 feral horses on Sable Island, Nova Scotia, Canada, in 2014 to test for the influence of host (age, sex and reproductive/social status) and environmental (location, local density and social group membership) factors on variation. Twenty-five strongyle species were identified, with individual infections ranging from 3 to 18 species with a mean richness (±1 s.d.) of 10.8 ± 3.1. Strongyle eggs shed in faeces were dominated by small strongyle (cyathostomins) species in young individuals, transitioning to large strongyles (*Strongylus* spp.) in adults. Egg counts were highest in young individuals and in the west or centre of the island for most species. Individuals in the same social group had similar parasite communities, supporting the hypothesis that shared environment may drive parasite assemblages. Other factors such as local horse density, sex, date and reproductive/social status had minimal impacts on infection patterns. This study demonstrates that mixed infections can be dynamic across host ontogeny and space and emphasizes the need to consider species-specific infection patterns when investigating mixed infections.

## Graphical abstract

**Figure:**
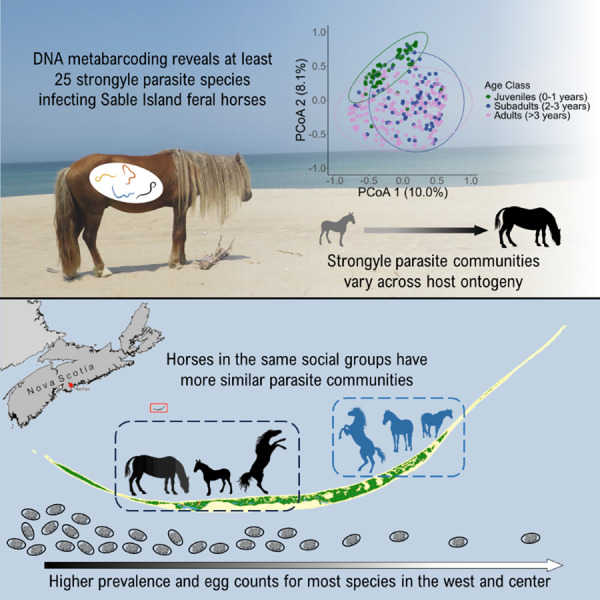

## Introduction

Understanding the ecology and evolution of host–parasite interactions requires knowledge of the intrinsic and environmental factors that drive among-individual variation in infection (Watson, 2013). This includes, for example, assessing the relative contributions of host age (Dobson and Pacala, 1988), genetics (Bishop, 2012), local density (Patterson and Ruckstuhl, 2013) and environmental heterogeneity (Pullan *et al*., 2012) on variation in infection status. However, despite concurrent infections by multiple parasite species (herein called ‘mixed infections’) being the norm (Bordes and Morand, 2009), most research in this field has so far focussed on single-species infection or indiscriminate aggregate measures of infection (e.g. fecal egg counts of major parasite clades). A greater consideration of mixed infections is important since co-occurring parasite species can have unique consequences not apparent in single infections (Hoarau *et al*., 2020). For example, variation in the composition of mixed infections can alter infection pathology (Ezenwa *et al*., 2021), and may result in unexpected epidemiological patterns such as increased transmission (Lass *et al*., 2013). Identifying factors driving variation in mixed infections is therefore essential to accurately assess their ecological and evolutionary consequences.

While still limited, literature on the role of intrinsic and extrinsic factors in shaping variation in mixed infections is beginning to emerge. Host age is hypothesized to play a key role, with younger individuals often having higher prevalence and abundance of certain parasite species (e.g. Parascaris spp. in horses (Fabiani *et al*., 2016); Trichostrongylus spp. in Soay sheep (Sinclair *et al*., 2016)) if juveniles have yet to develop acquired resistance (Fabiani *et al*., 2016; Sinclair *et al*., 2016). Similarly, variation between sexes has been documented in multiple host species (Bucknell *et al*., 1995; Santoro *et al*., 2014; Sweeny *et al*., 2022), possibly due to physiological differences, such as testosterone depressing immune responses in males (Ezenwa *et al*., 2012), or higher reproductive investment in females (Houdijk, 2008; Sweeny *et al*., 2022). Mixed infections can also vary across space in response to mechanisms such as dispersal limitation (Moss *et al*., 2020), propensity for certain climatic regions (Abbas *et al*., 2023), or sympatry among host species (Avramenko *et al*., 2018). Furthermore, mixed infection can vary across temporal scales, with seasonal patterns of parasite egg shedding driven by species-specific variation in life histories (Sargison *et al*., 2022; Sweeny *et al*., 2022), or as a consequence of priority effects that alter interactions among parasite species (Karvonen *et al*., 2019).

Despite increasing interest, testing hypotheses on the drivers of mixed infections remains challenging outside of clinical settings (Hoarau *et al*., 2020). Invasive sampling techniques to quantify parasite infection, such as kill-and-count or deworming treatment, are often difficult to implement for wild populations, especially in the case of longitudinal individual-based population studies, when hosts are elusive, or for populations of conservation concern (Aivelo and Medlar, 2018). Furthermore, invasive techniques are often resource intensive, which often limit conducting comprehensive surveys of parasite infections under field conditions. In contrast, non-invasive methods, such as fecal egg counts (FEC), are easier to employ, require fewer resources and limit negative impacts on study populations. However, traditional techniques relying on morphological features of eggs or larvae require significant training for accurate identification (Bradbury *et al*., 2022), or lack resolution due to limited differences in features among taxa (Aivelo and Medlar, 2018). Fortunately, advancements in DNA metabarcoding techniques now permit characterization of mixed parasitic infections using non-invasive samples (Aivelo and Medlar, 2018). In particular, amplification and sequencing of the ITS2 gene region using parasite DNA isolated from feces (commonly referred to as ‘nemabiome’ sequencing) has been utilized to characterize mixed parasite infections in multiple domestic (e.g. cattle; Avramenko *et al*., 2018, sheep; Redman *et al*., 2019, horses; Poissant *et al*., 2021) and wildlife (primates; Pafčo *et al*., 2018, reindeer; Davey *et al*., 2021, moose; Davey *et al*., 2023, roe deer; Beaumelle *et al*., 2022) species.

The need for parasite species distinction is particularly important for strongyle nematode infections of equids. There are at least 64 strongyle species (Family: Strongylidae) that infect the gastrointestinal tract of equines upon ingestion of parasite larvae from contaminated environments (Lichtenfels *et al*., 2008). Strongyle species are the most common parasite of horses and can vary in life history and pathology (Khan *et al*., 2015). Species in the *Strongylus* genus migrate through host tissue during larval development, with *S. vulgaris* notable for causing severe inflammation and thrombosis in the mesenteric artery that can be fatal (McCraw and Slocombe, 1976). Cyathostomins (Subfamily: Cyathostominae) do not migrate but encyst directly in the large intestine, and may cause larval cyathostominosis, a highly fatal syndrome associated with mass emergence of encysted larvae (Corning, 2009). Necropsy and deworming studies in horses have shown that the composition of mixed strongyle infections can vary across ontogeny (Steuer *et al*., 2022; Boisseau *et al*., 2023*a*), between sexes (Sallé *et al*., 2018) and environments (Saeed *et al*., 2019). Recent studies applying nemabiome sequencing in domestic horses further indicate that mixed strongyle infections may be influenced by reproductive status and climatic zones (Abbas *et al*., 2023) and vary across months (Poissant *et al*., 2021; Sargison *et al*., 2022). Furthermore, the composition of strongyle infections were found to be similar among 2 sympatric equine species (plains zebra and Grevy's zebra), emphasizing the influence of shared environment for generalist parasites such as strongyles (Tombak *et al*., 2021). However, the nemabiome approach has yet to be applied in a population of wild/feral equines to investigate drivers of among-individual variation in infections in the wild, limiting an accurate assessment of how this variation can influence the health of free-living equine populations.

The feral population of horses on Sable Island, Nova Scotia, Canada, provides a unique model for studying the ecology and evolution of host-parasite infections in the wild. The population has been subject to a long-term individual-based study since 2007 (Debeffe *et al*., 2016; Gold *et al*., 2019). Strict limits on human intervention mean that this population has never been given anthelmintics or veterinary care, providing a unique opportunity to study mixed infections under natural conditions. Previous studies have shown that Sable Island horses have higher strongyle fecal egg counts (FECs) relative to domestic horse populations (Debeffe *et al*., 2016). Furthermore, a general decline in FEC is observed across horse age (Debeffe *et al*., 2016), though older individuals shed higher proportions of *Strongylus* spp. eggs based on fecal cultures (Jenkins *et al*., 2020). Mean FEC was higher in female horses (1287 EPG) than males (1043 EPG). Individuals with higher energy requirements, such as lactating females (1437 EPG) had higher mean FEC than non-lactating females (672 EPG), though, there were no clear differences in mean FEC between bachelor males (916 EPG) and band stallions (898 EPG) (Debeffe *et al*., 2016). Finally, strongyle FEC was shown to vary along the length of the island, with FEC declining from west to east, possibly associated with denser vegetation and greater availability of permanent freshwater ponds in the west and center of the island (Debeffe *et al*., 2016). However, our current understanding of the ecology of strongyle infections in this population lacks species-specific resolution, particularly for the non-*Strongylus* species that constitute most of the species diversity (Poissant *et al*., 2021).

In this study, extensive host and environmental data was combined with nemabiome sequencing to identify factors associated with mixed infection composition and species-specific prevalence and FEC in the Sable Island horse population. Since age is associated with significant physiological change and strongyle FEC (Debeffe *et al*., 2016), we predicted that mixed infections would vary across ontogeny. Specifically, we expected younger individuals that have yet to develop acquired resistance to have higher strongyle diversity and FEC. Similarly, energy-limited individuals were predicted to have more diverse and abundant infections due to physiological differences in sex or reproductive status. Finally, given that strongyle species are environmentally transmitted, we expected individuals sharing the same space to have more similar infections.

## Materials and methods

### Study site and host population

**Figure 1.**
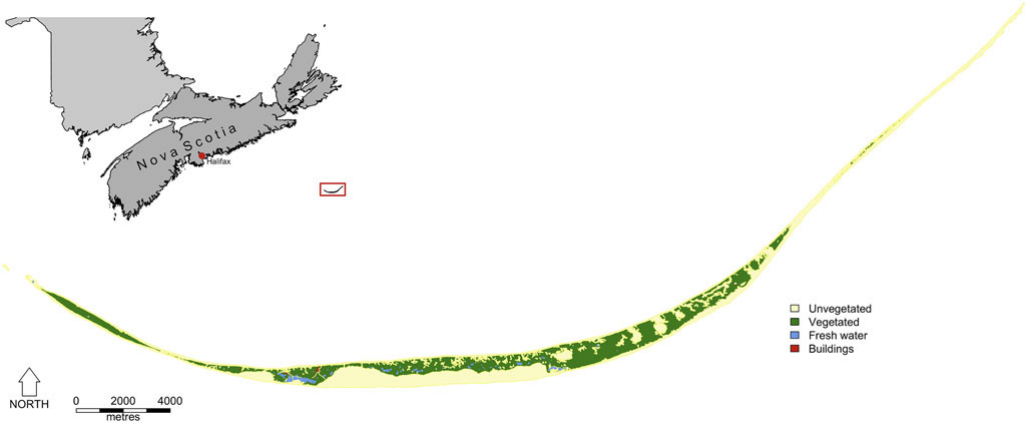
Map of Sable Island National Park Reserve, Nova Scotia, Canada (from Gold *et al*., 2019).

Sable Island National Park Reserve (43°55′N; −60°00′W) is a crescent-shaped sandbar 49 km long and 1.2 km wide at its widest point, located approximately 175 km off the east coast of Nova Scotia, Canada (Fig. 1). The island is home to a population of feral horses introduced in the 18th century which has been protected from human interference since 1961, including any handling, veterinary care, or supplemental feeding (Frasier *et al*., 2016). Since 2007, annual surveys have been conducted during the late breeding season (July–September) as part of a long-term individual-based study (Gold *et al*., 2019; Regan *et al*., 2020). Each day (weather permitting), 1 of 7 sections of the island was surveyed, resulting in complete coverage of the island approximately once a week, and multiple times over a field season. Horses were approached on foot and their appearance (coat color, markings, etc.), age class (e.g. foal, yearling, adult), sex, social band membership, time of observation and location within 5 m using a GPS recorded. Photographs of each horse were also taken to facilitate subsequent identification through comparison with a comprehensive photographic database.

As the study started in 2007, individuals born since 2006 could be aged accurately since foals and yearlings are easily distinguishable. Individuals born prior to 2006 were pooled in a single cohort assumed to be born in 2005. Given that most foals are born prior to the start of the field season, the exact birth date of individuals is unknown. Thus, we defined age of individuals in years, with foals defined as young-of-the-year (0 years old), increasing by 1 year every subsequent field season the individual was observed (e.g. yearlings have an age of 1 year). We expect most foals to be born in spring, thus, most foals are likely 0–4 months old at the start of the field season. The center of an individual's home range was inferred using the median longitude of all sightings for that individual, and local horse density associated with an individual was calculated as the total number of horses, excluding foals, whose median location was within an 8000-m radius of the individual's median location; this corresponds to the area used by horses for foraging (Marjamäki *et al*., 2013; Debeffe *et al*., 2016). Since strongyle parasites are transmitted through contaminated vegetation, local horse density was calculated as a function of total vegetated area (Marjamäki *et al*., 2013).

The social system of the horse is characterized by female-defense polygyny. Breeding groups (bands) are typically comprised of an adult male (stallion), breeding females (mares) and juveniles/subadults, although on some occasions multi-male bands do occur (i.e. a subordinate adult male may be present) (Contasti *et al*., 2012). Bands are generally stable throughout the duration of the field season, with most social dispersal occurring overwinter (Marjamäki *et al*., 2013). Unattached ‘bachelor’ males often associate in groups that are more ephemeral but still relatively stable (McDonnell and Murray, 1995). Hence, social groups for bachelors were defined as the unique grouping of individuals that were most often observed together over the field season. If a bachelor was observed in multiple social groupings an equal number of times, it was arbitrarily assigned to the largest social group. Female reproductive status was inferred by the presence or absence of a nursing foal (young-of-the-year), while male social status was defined as either band stallion (tending a harem), subordinate (in a band but not the dominant stallion), or bachelor.

### Parasite sample collection

Freshly dropped feces linked to specific individuals were collected either opportunistically during surveys or by targeted observation using disposable nitrile gloves, with one subsample typically stored on icepacks and another at ambient temperature. Special attention was given to avoid environmental contamination (i.e. sand or vegetation) and multiple areas of a fecal mass was sampled, whenever possible. Samples for this study were collected between July 23 and September 5, 2014.

Strongyle abundance for each fecal sample was measured as fecal egg counts (FEC), which were conducted on the day of collection using 4 ± 0.1 g of feces and 26 mL Sheather's sugar solution (specific gravity of 1.27) following a previously reported modified McMaster protocol (Debeffe *et al*., 2016). Counts were multiplied by 25 to calculate strongyle abundance in units of eggs per gram of feces (EPG). FECs were typically, but not always, performed using feces kept cool on icepacks, although earlier research indicated that FECs are not impacted by storage temperature in the field (Debeffe *et al*., 2016).

For each fecal sample, a strongyle L3 coproculture was also conducted following methods described in Poissant *et al*. (2021). Briefly, 30 ± 1 g of feces kept at ambient temperature was mixed with 3 heaped teaspoons of vermiculite and moistened with tap water in a glass tumbler. Coprocultures were covered with a petri dish and placed in a 26°C incubator for 6 days, misted with tap water and randomly shuffled in the incubator every other day. On the evening of the sixth day, a modified Baermann ‘glass-over-petri-dish’ technique (Roberts and O'Sullivan, 1950) was performed, with L3 larvae harvested the following morning. While species may respond differently to culture conditions, experimental evidence indicates that most eggs incubated at 26°C will reach the infective L3 stage by 3–4 days (Mfitilodze and Hutchinson, 1987) and that eggs of different species should have relatively similar hatching rates (Rupasinghe and Ogbourne, 1978). Harvested L3 larvae were fixed in 1–2 mL of 70% ethanol, stored at −20°C in the field and then at −80°C upon return to the mainland.

### DNA preparation and sequencing

A total of 731 larvae samples were collected from 504 individuals in 2014 (population size was 552). For this study, we randomly selected one sample from 320 different individuals. Samples were processed using protocols described in Poissant *et al*. (2021). The total number of L3 larvae per sample was estimated from the average worm count of two 5 μL aliquots, and approximately 2500 larvae were aliquoted. Aliquots were centrifuged at 14 000 RPM for 2 min and most of the ethanol removed, followed by the addition of 900 μL of lysis buffer (recipe in Avramenko *et al*., 2015). This process was repeated 2 more times to remove as much ethanol as possible and L3 were finally resuspended in 300 μL of lysis buffer. Larvae were then incubated at 95°C for 15 min and frozen at −80°C for a minimum of 12 h to encourage tissue lysis. Nine microlitre of proteinase K (20 mg mL^−1^) were then added and samples incubated at 50°C on a shaking incubator (300 rpm) until L3 were no longer visible (12–24 h). Proteinase K was then deactivated by incubating samples at 95°C for 15 min and a 1:10 dilution made using molecular grade water and stored at −80°C until further processing.

When larval samples had less than 2500 L3 larvae, the entire sample was processed (*n* = 14/320, range: 750–2400 L3). Excluding these samples from analyses did not quantitatively or qualitatively impact results, thus, these samples were retained for subsequent analyses.

Sequencing library preparation followed protocols described in Avramenko *et al*. (2015). Briefly, the ITS2 region was amplified in a first polymerase chain reaction (PCR) using the NC1 and NC2 primers (Gasser *et al*., 1993) with up to 3 random bases preceding the priming sequence and an adapter sequence to allow incorporation of barcodes in a subsequent PCR. Thermocycling consisted of 95°C for 3 min, followed by 25 cycles of 98°C for 20 s, 62°C for 15 s, 72°C for 15 s and a final extension at 72°C for 2 min. PCR products were purified using AMPure XP magnetic beads (Beckman Coulter, USA) at a ratio of 1:1 followed by gel electrophoresis to confirm successful amplification. A second PCR was performed using the initial PCR product to add unique combinations of indexes using the following thermocycler conditions: 98°C for 45 s, followed by 7 cycles of 98°C for 20 s, 63°C for 20 s and a final extension at 72°C for 2 min. Amplification was followed by a second round of magnetic bead purification. DNA concentration was quantified using a microvolume spectrophotometer and samples were pooled into libraries with approximately 100 ng of DNA per sample. The total concentration of DNA in the libraries was quantified with real-time PCR using the Universal KAPA Library Quantification Kits (Kapa Biosystems, USA), followed by sequencing on an Illumina MiSeq sequencer using 12 pM libraries with 20% PhiX sequencing control (Illumina, USA). The 320 samples considered in this study were sequenced across 3 separate runs; 2 used a v2 500-cycle Reagent Kit (*n* = 24, 15) and 1 used a v3 600-cycle Reagent Kit (*n* = 281) (Illumina, USA). Pooled libraries for the 2 v2 500-cycle kits included additional samples from Sable Island horses (repeat samples from the same individuals not considered herein), other horse populations, as well as cattle, bison and domestic sheep. The library for the v3 600-cycle kit contained only Sable Island horse samples.

### Bioinformatics pipeline

Each sequencing run was independently analyzed through an in-house bioinformatics pipeline (Poissant *et al*., 2021; pipeline version 2 available at https://data.mendeley.com/datasets/vhyysw8xt2/2) with minor modifications. First, as different sequencing kits result in different read lengths, reads obtained from the v3 600-cycle kit were trimmed to a maximum length of 250 bp prior to analyses using the DADA2 function *filterAndTrim* with the parameter *truncLen* set to 250 to parallel read lengths generated from v2 500-cycle kits. Primers and adapters were then removed using CutAdapt v4.1 (Martin, 2011), followed by processing through DADA2 v1.26 (Callahan *et al*., 2016). The variable lengths of the ITS2 gene region for equine strongyles results in species-specific variation in the length of overlap when merging paired reads (Poissant *et al*., 2021). To avoid a filtering bias against species with a shorter ITS2 gene region which have a longer overlapping region and a higher likelihood of random errors detected during merging, the maximum number of mismatches permitted during merging was based on percent mismatch (relative to the length of the overlapping region) rather than an absolute number of mismatches. For each sequencing run, a mismatch threshold of 2.5% was empirically identified as suitable to retain relatively abundant amplicon sequence variants (ASV) while limiting rare sequences containing an excessive number of errors. Following processing through DADA2, ITSx v1.1.3 (Bengtsson-palme *et al*., 2013) was used to remove the conserved gene regions flanking the ITS2, and taxonomy assigned to resulting ASVs using a curated ITS2 reference database (details below) and the DADA2 *assignTaxonomy* function with assignment confidence set to ≥80%. To account for possible index hopping, any species that represented less than 1/2500 of the proportion of reads in a sample was removed (i.e. ~1 larvae out of the 2500). Non-equine strongyle sequences that were present in mixed sequencing runs, but not in the run containing only Sable Island horse samples, were removed assuming these species were likely due to index hopping from other host species. Finally, any sample with less than 2500 total reads were not considered for analyses, to ensure sequencing depth was sufficient to represent the parasite communities.

### ITS2 reference database

Version 1.4.0 of the Nematode ITS2 Sequence Database (Workentine *et al*., 2020) was obtained from www.nemabiome.ca/its2-database.html. ITS2 sequences of strongyle (family Strongylidae) nematode parasites of equines (family Equidae) were further curated replicating the steps taken in Poissant *et al*. (2021). First, individual species with various synonymous names were renamed to a common name following Lichtenfels *et al*. (2008). Of the 323 equine strongyle ITS2 sequences present in the database, 40 incomplete sequences were identified following alignment using MAFFT v7.490 (Katoh *et al*., 2002) and visual inspection using UGENE v42 (Okonechnikov *et al*., 2012) and removed. To identify potential errors in species identification for GenBank entries, IQ-TREE v.1.6.12 (Nguyen *et al*., 2015) was used to construct a phylogenetic tree using maximum likelihood approaches using the remaining 283 sequences. Similar to Poissant *et al*. (2021), one sequence did not cluster with other sequences of the same species and was removed (accession number: Y08619.1; *Cyathostomum catinatum*). Ultimately, 282 sequences remained, representing 43 of the 64 equine strongyle species recognized in Lichtenfels *et al*. (2008), with 1 to 44 sequences available per species. Version 1.4.0 contains 18 new sequences compared to version 1.3.0 used in Nielsen *et al*. (2022), but no new species. Only ITS2 sequences of strongyle species described in Lichtenfels *et al*. (2008) were curated following the described steps. Any other ITS2 sequences from the nematode database were left unchanged, even if they are known to infect horses.

### Estimating correction factors

Nemabiome sequencing may result in biased species relative proportions due to species-specific variation in the number of cells, copy numbers of the ITS2 gene region and PCR efficiency (Avramenko *et al*., 2015). To account for this, Avramenko *et al*. (2015) sequenced mock communities of morphologically identified L3 to estimate correction factors for cattle strongyles. Due to the inability to create mock communities for equine strongyles, Poissant *et al*. (2021) compared the relative proportions of L3 species identified morphologically (*S. vulgaris, S. equinus and S. edentatus*, non-*Strongylus* species) in field samples with the proportion of reads assigned to those species using nemabiome sequencing. However, species-specific biases (estimated as the regression between molecular and morphological relative proportion) reported in Poissant *et al*. (2021) do not take into consideration that the observed proportion of reads assigned to a given species is a function of both the morphological:molecular bias for that species as well as those of other species present. For example, if bias results in one species being over-represented in a sample, the relative proportion of other species will decrease as a result.

To obtain more accurate estimates of correction factors, subsamples of L3 larvae fixed in 10% formalin were morphologically identified replicating methods in Poissant *et al*. (2021). Across 57 paired samples, a total of 70 489 larvae were identified, ranging from 102 to 6370 larvae per sample (median = 954). Due to limited distinguishing features among strongyle L3 larvae, morphological identification (according to Russell, 1948) was only possible for certain species including *Strongylus vulgaris* and *S. equinus.* Larvae containing 16 or more distinct intestinal cells, exclusive of those identified as *S. vulgaris* and *S. equinus*, were considered as *S.edentatus*, as molecular results indicated low prevalence and abundance of other candidate Strongylin species. Although a few additional species and genera could be identified, (e.g. *Gyalocephalus capitatus* and *Poteriostomum spp.*), low prevalence and abundance (both morphological and molecular) precluded accurate estimation of their correction factors. Ultimately, L3 were morphologically classified as either 1 of the 3 *Strongylus* species or as non-*Strongylus*.

Molecular species classifications were grouped to parallel morphological classifications. Based on a maximum-likelihood phylogenetic tree, unclassified ASVs (which represented ASVs with less than 80% confidence in species assignment) clustered with non-*Strongylus* species and were grouped together. Correction factors were estimated for the 3 *Strongylus* species and the non-*Strongylus* group simultaneously using the *nls* function in R using the following formulation:
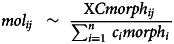
where *mol_ij_* and *morph_ij_* are row vectors of molecular and morphological proportions for each taxon *i* in each sample *j*, respectively, *X* is a design matrix (of 0s and 1s) linking each element of *morph_ij_* to the appropriate element of *C*, a column vector containing taxon-specific correction factors for each taxa, and *c_i_* and *morph_i_* are taxon-specific correction factors and row vectors of morphological proportions in each sample for all *n* taxon, respectively. To allow for estimating multiple correction factors simultaneously, the correction factor for non-*Strongylus* was set to 1. As such, resulting correction factors for *Strongylus* species reflect how many reads these species generate per L3 larvae relative to non-*Strongylus* species. The correction factors for *S. edentatus*, *S. equinus* and *S. vulgaris* relative to non-*Strongylus* species were estimated to be 1.523, 1.809 and 4.658, respectively, indicating that all *Strongylus* species yielded more reads per L3 larvae than non-*Strongylus* species. To correct molecular proportions for *Strongylus* species, the number of reads assigned to each species were divided by the correction factors prior to calculating relative proportions. Sample FEC were then multiplied with the relative proportion of each parasite species to estimate species-specific FEC. One sample had zero EPG, but larvae were successfully harvested from the coproculture, thus a minimum of one egg was assumed for the FEC (25 EPG).

### Statistical analyses

#### Species diversity and species-specific analyses

Species diversity of an individual's nemabiome was measured across various univariate indices: species richness, Inverse Simpson diversity index and Shannon's diversity index (normalized, ranging from 0 to 1). Diversity indices were calculated using the *diversity* function in the R package ‘vegan’ 2.6–4 (Oksanen *et al*., 2022). The Inverse Simpson index places greater weight on species evenness (dominance of certain species), while Shannon's index considers both species richness and evenness.

To test which host and environmental factors were associated with species diversity indices, species-specific prevalence and species-specific FEC, generalized linear models (GLMs) were fitted using *glmmTMB* in the R package ‘glmmTMB’ v1.1.6 (Brooks *et al*., 2023). A global model was constructed which included: age (3rd order orthogonal polynomial), sex (2 level factor), median longitude (standardized to a mean of 0 and s.d. = 1, 2nd order orthogonal polynomial), an interaction between linear age and linear longitude, ordinal date (continuous; standardized to a mean of 0 and s.d. = 1) and local horse density (continuous). The model included age^2^ and age^3^ to account for possible curvilinear relationships between age and parasite infection, for example, if resistance peaks or dips during certain life-stages (e.g. immunosenescence Froy *et al*., 2019). Longitude and the interaction between age and longitude, found to explain strongyle FEC in earlier research (Debeffe *et al*., 2016), were also included in addition to longitude^2^ to test for possible non-linear spatial patterns. The species richness models used a Poisson probability distribution (*family* *=* *poisson*), while the Inverse Simpson and the Shannon's diversity index models used a Gaussian probability distribution (*family* *=* *gaussian*). Prevalence was modeled using binomial models (*family* *=* *binomial*), while species-specific FECs were cube-root transformed and modeled using zero-inflated GLMs using the linear parameterization of the tweedie probability distribution (*ziformula* *=* *~1, family* *=* *tweedie*) to account for overdispersion common in parasite abundance measures (Wilson *et al*., 1996). Species-specific models were limited to parasite species with at least 10 positive samples, resulting in a total of 19 species for which prevalence and FEC were modelled.

A multi-model inference approach was used for model selection (Burnham and Anderson, 2002). Starting with the global model described above, all possible combinations of fixed effects were constructed and ranked by AIC_c_, and conditional model averaging with models with ΔAIC_c_ <2 was implemented using the R package ‘MuMIn’ v1.47.5 (Bartoń, 2023) to calculate parameter estimates. Prevalence and FEC for certain species could not be model-averaged (i.e. global models could not converge if prevalence is too high/low, thus AIC weights could not be calculated). For those cases, the model with the lowest AIC_c_ was chosen for interpretation. Finally, a Spearman correlation test was used to test if species-specific prevalence and FEC were associated.

#### Community analyses

Jaccard and Bray-Curtis dissimilarity matrices were calculated using species-specific FEC to quantify differences in mixed infection composition between samples using the *vegdist* function from the *vegan* package (Oksanen *et al*., 2022) and visualized using a principal coordinate analysis (PCoA). Jaccard dissimilarity only considers species presence–absence, while Bray–Curtis dissimilarity incorporates species presence–absence and their abundance. To test which host and environmental factors were associated with infection species composition, a permutational analysis of variance (PERMANOVA) was conducted on the Jaccard and Bray–Curtis distances using the *adonis2* function in the *vegan* package with 9999 permutations (Oksanen *et al*., 2022). Parameters in the PERMANOVA included: age (3rd order polynomial), sex (2-level factor), median longitude (2nd order polynomial, standardized to a mean of 0 and s.d. = 1), ordinal date (continuous; standardized to a mean of 0 and s.d. = 1) and local horse density (continuous). Foals (age 0) were identified to have significantly higher variation in species composition relative to other age groups (i.e. greater beta dispersion). As PERMANOVA is unable to distinguish if significant associations are due to variation within-groups or between-groups, a second PERMANOVA was conducted excluding foals to meet the assumption of multivariate homoscedasticity. Given that the no-foal PERMANOVA had near identical results to the full model, the interpretations and visualizations are based on the full model. To simplify visualization of the PCoA, individuals were grouped into age classes: juveniles (age 0–1), subadults (age 2–3), adults (age >3). To confirm that visualizations and the PERMANOVA had consistent interpretations regardless of how age was classified, 2 additional PERMANOVAs were constructed in which age (as a continuous variable) was replaced with (1) age class (3 levels) or (2) age as a categorical variable (in years, 10 levels). Given that all models were quantitatively and qualitatively similar, only the PERMANOVA with age as a continuous variable is presented.

To test for the effects of reproductive and social status on strongyle community composition, PERMANOVAs were conducted on an adult-only (age >3) subsets of the data, with social status (males: bachelor or band stallion) or reproductive status (females: with foal or without a foal) replacing sex. Subsequently, sex-specific adult-only PERMANOVAs were conducted to further investigate differences (Supplementary Materials). Finally, a univariate PERMANOVA was conducted to estimate how much variation in parasite communities was attributed to social group.

## Results

### Sequencing outputs, quality filtering and taxonomic assignment

Sequencing resulted in 5838–84 704 read pairs per sample (35 990 ± 9742 [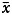 ± 1 s.d.]). Following filtering and processing, between 3264 and 33 673 amplicons remained per sample (15 657 ± 4376 [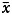 ± 1 s.d.]). The proportions of reads with taxonomy assigned were high, with most samples having less than 2% unclassified reads and only 6 samples having more than 10% (maximum: 15.4%). The majority of unclassified ASVs were assigned to *Cylicostephanus calicatus* and *Cylicocyclus ashworthi* at a confidence level below the 80% threshold.

### Alpha diversity

**Figure 2.**
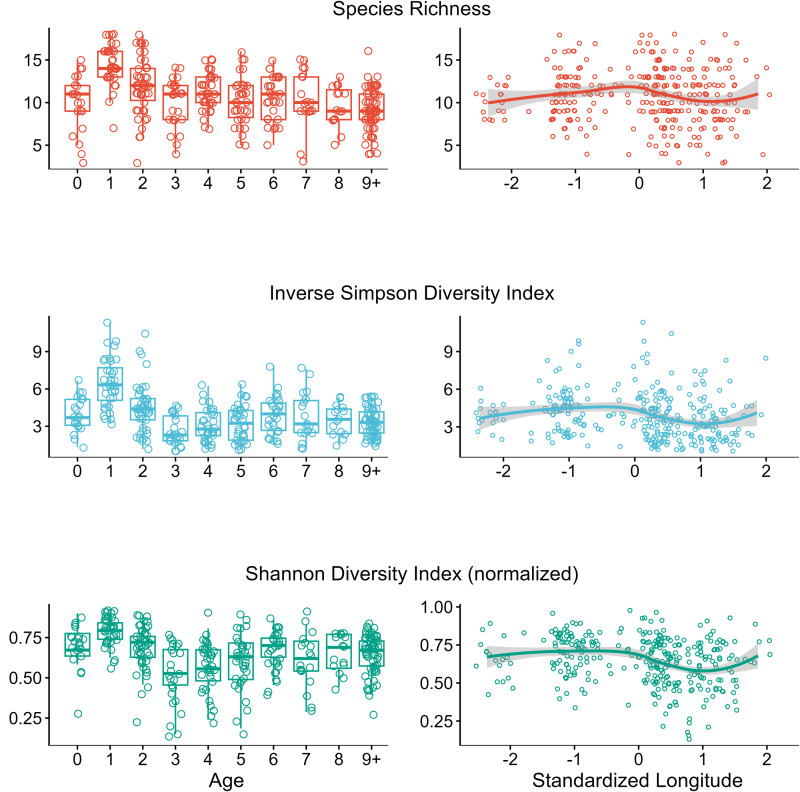
Strongyle parasite species richness, Inverse Simpson diversity index and Shannon diversity index for 320 horses on Sable Island, Nova Scotia, Canada, across host age and standardized longitude. Each point indicates an individual sample. Boxplots on age plots show mean values for each age (in years). Lines in longitude plots describing the conditional means with a 95% confidence interval.

A total of 25 strongyle species were identified, with individual samples containing between 3 and 18 species with a mean richness (± 1 s.d.) of 10.8 ± 3.1. Species richness was highest in yearlings (14.7 ± 2.6), and significantly decreased with age (*P* < 0.01, Table 1) with the oldest individuals (age 9+) having the lowest richness (9.31 ± 2.5). Similarly, the Inverse Simpson and Shannon indexes decreased with horse age (*P* < 0.01, Table 1, Fig. 2). However, positive curvilinear age associations (age^2^, *P* < 0.01, Table 1) for both indexes suggest that the evenness of strongyle communities are higher in younger (i.e. 1–3 years old) and older adults (i.e. 8–9 years old) (Fig. 2). Furthermore, both Inverse Simpson and Shannon indexes decreased west to east, suggesting that horses in the east have lower diversity and evenness of strongyle parasite communities (*P* < 0.01, Table 1).

**Table 1.** Model averaging results (conditional) for alpha diversity measures of strongyle parasites communities of Sable Island horses (*n* = 320) in response to host and environmental factors

	Species richness				Inverse simpson diversity index				Shannon diversity index			
	Estimate	Adjusted SE	*z*	*P*	Estimate	Adjusted SE	*z*	*P*	Estimate	Adjusted SE	*z*	*P*
Intercept	**2**.**37**	**0**.**018**	**132**.**46**	**2 × 10^−16^**	**4**.**21**	**0**.**68**	**6**.**21**	**2 × 10^−16^**	**0**.**72**	**0**.**066**	**10**.**92**	**2 × 10^−16^**
Age	**−1.89**	**0**.**31**	**6**.**04**	**2 × 10^−16^**	**−10.95**	**1**.**69**	**6**.**47**	**2 × 10^−16^**	**−0.51**	**0**.**13**	**3**.**81**	**0.00014**
Age^2^	−0.12	0.31	0.40	0.69	**5**.**92**	**1**.**68**	**3**.**53**	**0**.**0042**	**0**.**65**	**0**.**13**	**4**.**81**	**1.5 × 10^−6^**
Age^3^	0.45	0.31	1.56	0.12	**–**	**–**	**–**	**–**	0.15	0.13	1.15	0.25
Longitude	−0.50	0.31	1.61	0.11	**−6.90**	**1**.**77**	**3**.**89**	**0**.**00010**	**−0.77**	**0**.**14**	**5**.**37**	**1.0 × 10^−7^**
Longitude^2^	−0.58	0.32	1.84	0.066	−3.21	1.73	1.86	0.063	−0.15	0.15	1.75	0.33
Age:longitude	−4.27	5.47	0.78	0.44	−8.94	29.30	0.31	0.76	1.23	2.32	0.53	0.60
Sex	0.020	0.035	0.59	0.55	0.21	0.19	1.13	0.26	0.010	0.015	0.68	0.50
Local horse density	**–**	**–**	**–**	**–**	**−0.035**	**0**.**026**	**1**.**33**	**0**.**18**	−0.0034	0.0019	1.75	0.080
Ordinal date	−0.020	0.17	1.14	0.26	−0.12	0.095	1.26	0.20	–	–	–	–

Models were considered for averaging if they had an ΔAIC_c_ <2. Dashes indicate fixed effects that were not present in any top model. Bolded rows indicate significant (*P* < 0.05) factors.

### Species-specific prevalence

**Figure 3.**
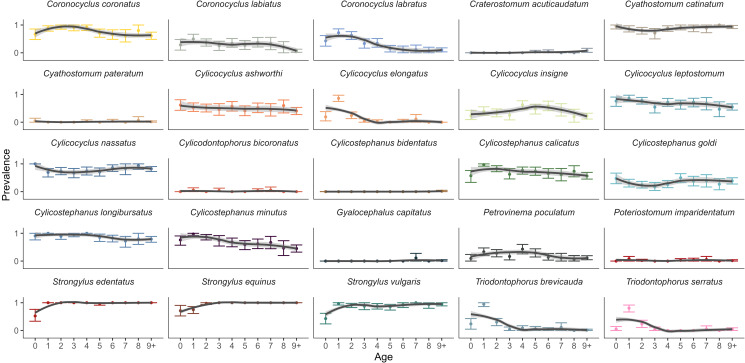
Prevalence of 25 strongyle species identified by DNA metabarcoding across host age for 320 Sable Island horses in 2014. Lines show the conditional means with a 95% confidence interval. Each point indicates the mean prevalence for each age with error bars estimated as 95% bootstrap confidence intervals.

**Figure 4.**
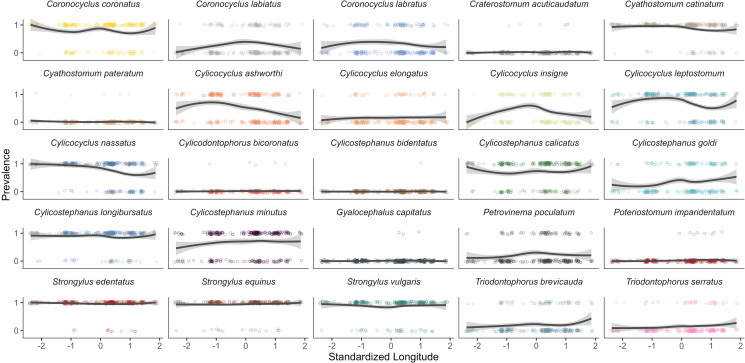
Prevalence of 25 strongyle species identified by DNA metabarcoding across a west to east gradient on Sable Island (standardized to a mean of 0, s.d. of 1) for 320 Sable Island horses in 2014. Each point indicates an individual sample, with lines describing the conditional means with a 95% confidence interval.

The prevalence of the 25 species of strongyles detected in Sable Island horses varied greatly (Table 2) with declines across horse age being the most common patterns followed by declines from west to east (Figs 3 & 4). At the population level, the 3 *Strongylus* species had the highest prevalence with *S. edentatus* found in 96.6% of the population, followed by *S. equinus* (95.0%) and *S. vulgaris* (89.1%). Non-migratory large strongyles had lower prevalence comparatively, with *Triopdontophorus brevicauda*, *T. serratus and Craterostomum acuticaudatum* found in 19.7%, 14.4% and 1.4% of samples, respectively. Seven small strongyle species had population-wide prevalence greater than 50%, with C*yathostomum catinatum* (88.1%), *Cylicostephanus longibursatus* (87.8%) and *Cylicocyclus nassatus* (78.4%) being the most common. Other species were less common, such as *Cylicocyclus insigne* (37.5%) and *Cylicocyclus elongatus* (15.9%). Five small strongyle species had a population prevalence of less than 3% (*n* < 9/320) (e.g. *Poteriostomum imparidentatum*, *Cyathostomum pateratum*), with *Cylicostephanus bidentatus* only found in a single sample.

**Table 2.** Population-wide prevalence and mean fecal egg counts (FEC) for 25 strongyle species identified using DNA metabarcoding in 320 horses sampled on Sable Island, Nova Scotia, Canada, in 2014

Species	Population prevalence (%)	Mean FEC (EPG ± 1 s.d.)	Prevalence rank in domestic horses^a^ (Bellaw and Nielsen, 2020)
*Strongylus edentatus*	96.56	311.75 ± 290.71	–
*Strongylus equinus*	95.00	545.77 ± 725.82	–
*Strongylus vulgaris*	89.06	41.11 ± 62.65	–
*Cyathostomum catinatum*	88.13	91.33 ± 151.89	1
*Cylicostephanus longibursatus*	87.81	120.11 ± 166.86	3
*Cylicocyclus nassatus*	78.44	117.81 ± 226.22	2
*Coronocyclus coronatus*	77.19	55.91 ± 94.75	5
*Cylicostephanus calicatus*	71.25	11.94 ± 16.98	6
*Cylicostephanus minutus*	68.13	70.23 ± 123.24	8
*Cylicocyclus leptostomum*	67.81	45.44 ± 82.62	7
*Cylicocyclus ashworthi*	49.38	12.82 ± 28.79	10
*Cylicocyclus insigne*	37.50	16.89 ± 41.87	9
*Cylicostephanus goldi*	33.75	4.88 ± 12.42	4
*Coronocyclus labratus*	31.56	8.18 ± 25.79	12
*Coronocyclus labiatus*	28.75	4.51 ± 12.98	13
*Petrovinema poculatum*	21.88	1.69 ± 5.54	15
*Triodontophorus brevicauda*	19.69	16.43 ± 56.15	–
*Cylicocyclus elongatus*	15.94	12.49 ± 50.67	17
*Triodontophorus serratus*	14.38	3.45 ± 11.70	16
*Poteriostomum imparidentatum*	2.50	0.10 ± 0.91	14
*Craterostomum acuticaudatum*	2.19	0.09 ± 0.71	–
*Cyathostomum pateratum*	1.56	0.05 ± 0.48	11
*Cylicodontophorus bicoronatus*	1.56	0.04 ± 0.36	18
*Gyalocephalus capitatus*	<1.94	0.01 ± 0.15	–
*Cylicostephanus bidentatus*	<1.31	0.01 ± 0.13	–

Prevalence rank of cyathostomin species in domestic horses inferred from the metanalysis of Bellaw and Nielsen (2020) is also shown.

a Strongylins and certain rare cyathostomin species were not included in Bellaw and Nielsen (2020), thus marked with dashes.

The relationship between prevalence and host age differed among strongyle species (Fig. 3). The prevalence of the *Strongylus* species was lowest in foals and stayed relatively stable following an increase from age 0 to 3 (Fig. 3), with *S. equinus* and *S. vulgaris* being the only species that significantly increased in prevalence across ontogeny (*P* < 0.05, Table 3). An increase in prevalence across age was also noted for *Cylicostephanus goldi*. Otherwise, the prevalence of most species (12/19 tested) declined as horse age increased (*P* < 0.05, Table 3; Fig. 3). Prevalence appeared to peak at certain ages for some species (negative coefficient for significant age^2^ associations), such as *Cylicocyclus insigne* and *Petrovinema poculatum* prevalences peaking at age 4 (61.7% and 46.8%, respectively; Fig. 2). In contrast, *Triodontophotus brevicauida, T. serratus* and *Cylicocyclus elongatus* had the opposite curvilinear trend with significant dips in prevalence in young adults following by an increase in older individuals. Furthermore, significant age^3^ associations were observed for 9 species, suggesting stabilization of prevalence in adults following a peak (e.g. *Cylicocyclus nassatus*) or dip (e.g. *Coronocyclus coronatus*) (*P* < 0.05, Table 3; Fig. 3).

**Table 3.** Model averaging results (conditional) for specie-specific prevalence of 19 strongyle parasite species found in Sable Island horses (*n* = 320) in response to host and environmental factors

Species	Intercept		Age		Age^2^		Age^3^		Longitude		Longitude^2^		Age:Longitude		Sex (M)		Local horse density		Ordinal date	
	Est.	*P*	Est.	*P*	Est.	*P*	Est.	*P*	Est.	*P*	Est.	*P*	Est.	*P*	Est.	*P*	Est.	*P*	Est.	*P*
*Strongylus edentatus*^a^	**5.89**	**<0**.**01**	**21**.**20**	**0**.**11**	**−33.42**	**<0**.**01**	–	–	–	–	–	–	–	–	–	–	–	–	–	–
*Strongylus equinus*^a^	**35.31**	**0**.**03**	**610**.**36**	**0**.**04**	172.33	0.06	–	–	8.20	0.12	–	–	–	–	–	–	–	–	–	–
*Cyathostomum catinatum*	1.54	0.23	**10**.**83**	**<0**.**01**	−1.79	0.64	**9**.**16**	**<0**.**01**	–	–	3.50	0.39	0.69	0.08	–	–	0.07	0.25	−0.13	0.53
*Strongylus vulgaris*	**2**.**33**	**<0**.**01**	6.71	0.08	3.88	0.29	**−7.67**	**0**.**04**	**−12.54**	**<0**.**01**	–	–	−0.24	0.51	−76.07	0.34	–	–	**0**.**39**	**0**.**035**
*Cylicostephanus longibursatus*	**2**.**33**	**<0**.**01**	**−12.23**	**<0**.**01**	−1.73	0.60	5.06	0.12	−4.92	0.15	1.04	0.75	0.15	0.68	35.13	0.59	−0.03	0.43	0.096	0.59
*Cylicocyclus nassatus*	**1**.**99**	**0**.**04**	–	–	4.20	0.15	**−8.63**	**<0**.**01**	**21**.**26**	**<0**.**01**	6.62	0.16	−0.12	0.68	–	–	–	–	–	–
*Coronocyclus coronatus*	2.75	0.08	**−11.41**	**<0**.**01**	−4.95	0.04	**7**.**85**	**<0**.**01**	**−6.67**	**<0**.**05**	6.42	0.13	0.13	0.67	−53.31	0.26	−0.07	0.12	−0.22	0.14
*Cylicostephanus calicatus*	**3**.**82**	**<0**.**01**	**−8.20**	**<0**.**01**	−2.52	0.28	2.45	0.29	−1.43	0.62	**8**.**56**	**0**.**02**	0.20	0.45	**−101.58**	**0.02**	**−0.10**	**0**.**02**	−0.20	0.14
*Cylicocyclus leptostomum*	**0**.**87**	**0**.**01**	**−9.47**	**<0**.**01**	–	–	−0.85	0.72	**−9.34**	**<0**.**01**	−4.24	0.07	0.10	0.71	63.45	0.17	−0.01	0.78	−0.13	0.31
*Cylicostephanus minutus*	**1**.**01**	**0**.**03**	**−13.74**	**<0**.**01**	1.45	0.55	–	–	1.78	0.44	−2.66	0.24	−0.28	0.29	–	–	−0.03	0.33	−0.14	0.28
*Cylicocyclus ashworthi*	0.06	0.92	−4.52	0.05	–	–	−2.12	0.33	**−12.02**	**<0**.**01**	**−5.16**	**0**.**04**	−0.48	0.05	70.33	0.09	0.03	0.34	−0.17	0.18
*Cylicocyclus insigne*	−1.05	0.21	−2.17	0.36	**−8.19**	**<0**.**01**	−1.64	0.46	−0.88	0.73	**−11.24**	**<0**.**01**	0.37	0.13	–	–	0.04	0.25	–	–
*Cylicostephanus goldi*	**−0.75**	**<0**.**01**	**4**.**81**	**0**.**04**	2.16	0.32	**−4.54**	**0**.**04**	**7**.**73**	**<0**.**01**	–	–	–	–	35.93	0.38	–	–	**0**.**43**	**<0**.**01**
*Coronocyclus labratus*	−2.15	0.12	**−22.11**	**<0**.**01**	2.98	0.28	**8**.**69**	**<0**.**01**	−5.62	0.08	**−8.46**	**0**.**01**	0.13	0.65	−76.09	0.17	0.07	0.11	−0.089	0.54
*Coronocyclus labiatus*	**−1.21**	**0**.**01**	**−12.70**	**<0**.**01**	−4.69	0.07	−2.58	0.28	5.09	0.11	**−9.69**	**<0**.**01**	0.20	0.46	−79.50	0.17	0.02	0.66	**−0.53**	**<0**.**01**
*Petrovinema poculatum*	0.65	0.63	**−7.13**	**0**.**02**	**−5.79**	**0**.**03**	4.25	0.11	2.05	4.62	−1.51	0.62	0.21	0.47	–	–	−0.08	0.07	−0.082	0.56
*Triodontophorus brevicauda*	**−2.26**	**<0**.**01**	**−27.66**	**<0**.**01**	**9**.**87**	**0**.**03**	**9**.**86**	**0**.**01**	3.55	0.25	–	–	0.41	0.22	–	–	−0.02	0.69	−0.067	0.69
*Cylicocyclus elongatus*	**−2.97**	**<0**.**01**	**−34.72**	**<0**.**01**	**13**.**41**	**0**.**03**	**11**.**96**	**<0**.**01**	2.56	0.45	−2.28	0.49	0.45	0.23	–	–	−0.02	0.67	−0.37	0.052
*Triodontophorus serratus*	**−6.44**	**<0**.**01**	**−35.81**	**<0**.**01**	**51**.**13**	**<0**.**01**	**46**.**33**	**<0**.**01**	**8**.**65**	**0**.**048**	−5.78	0.28	0.64	0.11	−50.36	0.47	0.09	0.16	−0.065	0.76

Models were considered for averaging if they had an ΔAIC_c_ <2. Significant fixed effects are bolded (*P* < 0.05). Dashes indicate fixed effects that were not present in any top model.

a Indicates species which could not be model averaged due to difficulty in fitting a global model, for which the top models (ΔAIC_c_ = 0) was used for parameter estimates. Dashes indicate fixed effects not present in the top model for these species.

Prevalence was found to vary along the length of the island for certain species (Fig. 4). Five species decreased in prevalence from west to east, such as *Cylicocyclus nassatus*, which decreased from 90% in the west to 60% in the east (*P* < 0.01, Table 3, Fig. 4). In contrast, the prevalence of *Cylicostephanus goldi* and *Triodontophorus serratus* increased towards the east side of the Island (*P* < 0.01, Table 3). A few species exhibited curvilinear trends along the length of the island (i.e. significant longitude^2^ associations), with *Cylicocyclus ashworthi*, *Cylicocyclus* insigne, *Coronocyclus labratus* and *Coronocyclus labiatus* having significantly higher prevalence near the center (*P* < 0.05, Table 3). Only *Cylicostephanus calicatus* was less common in the center of the island (*P* = 0.02)

Ordinal date was significantly associated with 3 species, with *Cyathostomum catinatum* and *Cylicostephanus goldi* becoming more prevalent as the field season progressed, while *Coronocyclus labiatus* became less prevalent (*P* = 0.019, Table 1). *Cylicostephanus calicatus* was the only species to vary (negatively) with density (*P* < 0.05, Table 3) and age:longitude interactions (*P* = 0.02). Horse sex was not associated with the prevalence of any species.

### Species-specific fecal egg counts

Similar to prevalence patterns, species-specific FEC varied greatly among parasite species (Table 2). *Strongylus equinus* and *S. edentatus* had noticeably higher mean FEC (± 1 s.d.) relative to other species with 545.8 ± 725.8 and 311.8 ± 290.7 EPG, respectively. In contrast to the other *Strongylus* species, *S. vulgaris*, had a mean of 41.1 ± 62.7 EPG. Among the small strongyles, *Cylicostephanus longibursatus*, *Cylicocyclus nassatus* and *Cyathostomum catinatum* had the highest mean FEC (120.1 ± 166.9, 117.8 ± 226.2 and 91.3 ± 151.9 EPG, respectively). Most species had much lower mean FEC, typically <20 (Table 2). Patterns of species-specific FEC closely paralleled prevalence, with higher FEC associated with higher prevalence (Spearman's rank correlation coefficient = 0.93, *P* < 0.0001), though, the highly prevalent *Cylicostephanus calicatus* (prevalence = 71.3%) had relatively low mean FEC (11.9 ± 17.0 EPG).

**Figure 5.**
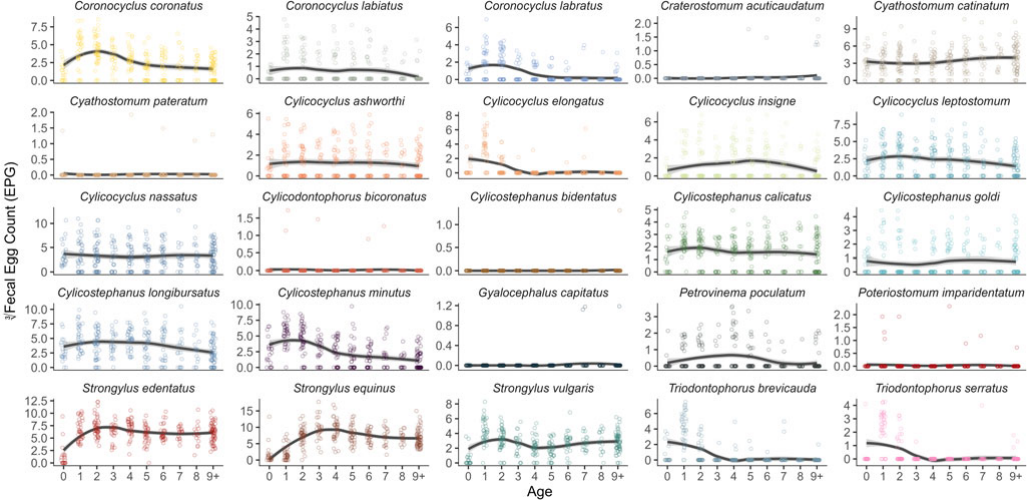
Fecal egg counts (FEC) of 25 strongyle species across host age for 320 Sable Island horses in 2014. Species-specific FEC was calculated by multiplying aggregate strongyle FEC by species-specific relative abundance estimated from DNA metabarcoding. Each point indicates an individual sample, with lines describing the conditional means with a 95% confidence interval. Note that independent y-axis were used per species to better visualize trends.

**Figure 6.**
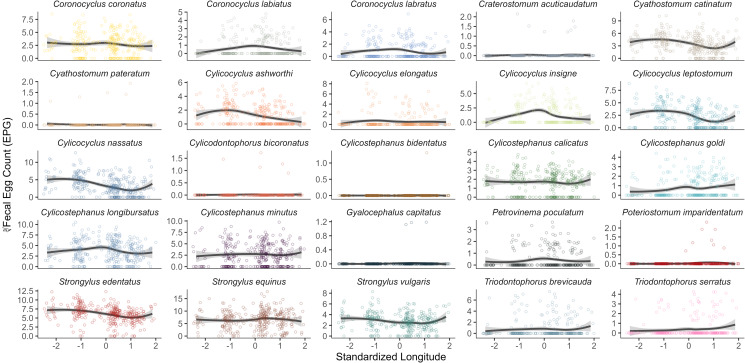
Fecal egg counts (FEC) of 25 strongyle species identified by DNA metabarcoding across a west to east gradient on Sable Island (standardized to a mean of 0, s.d. of 1) for 320 Sable Island horses in 2014. Species-specific FEC was calculated by multiplying aggregate strongyle FEC by species-specific relative abundance estimated from DNA metabarcoding. Each point indicates an individual sample, with lines describing the conditional means with a 95% confidence interval. Note that independent y-axis were used per species to better visualize trends.

There was clear variation across age for *Strongylus* FEC, with foals and adults having the lowest FEC relative to yearlings and subadults (Fig. 5). Species-specific FEC of non-migratory large strongyles exhibited patterns similar to those seen with prevalence (Figs 5 & 6), with yearlings having the highest FEC for the *Triodontophorus brevicauda* (145.6 ± 143.6 EPG) and *T. serratus* (26.7 ± 24.5 EPG, age = 1). For 9 species, FEC significantly decreased with age (Table 4), whereas only *Strongylus equinus* and *Cyathostomum catinatum* showed an increase. Negative age^2^ associations were identified for 6 species indicating an increase in FEC, typically peaking between 1 and 3 years old, followed by a decline in individuals >3 years-old (Fig. 5). Conversely, 4 species had significant positive associations with age^2^, suggesting that younger and older adults had higher FEC of these species (Fig. 5). Finally, most species tested (11/19) had significant positive associations with age^3^ suggesting that for most species, FEC peaks in younger individuals, followed by stabilization into adulthood (Fig. 5).

**Table 4.** Model averaging results (conditional) for specie-specific fecal egg counts of 19 strongyle parasite species found in Sable Island horses (*n* = 320) in response to host and environmental factors. Models were considered for averaging if they had an ΔAIC_c_ <2

Species	Zero-Inflation		Intercept		Age		Age^2^		Age^3^		Longitude		Longitude^2^		Age:Longitude		Sex (M)		Local horse density		Ordinal date	
	Est.	*P*	Est.	*P*	Est.	*P*	Est.	*P*	Est.	*P*	Est.	*P*	Est.	*P*	Est.	*P*	Est.	*P*	Est.	*P*	Est.	*P*
*Strongylus edentatus*	**−3.37**	**<0**.**01**	**1**.**83**	**<0**.**01**	–	–	**−1.71**	**<0**.**01**	**1**.**99**	**<0**.**01**	**−2.13**	**<0**.**01**	−0.31	0.31	–	–	−0.05	0.15	<0.01	0.50	−0.020	0.27
*Strongylus equinus*	**−5.02**	**<0**.**01**	**1**.**82**	**<0**.**01**	**4**.**62**	**<0**.**01**	**−6.80**	**<0**.**01**	**4**.**24**	**<0**.**01**	–	–	−0.22	0.63	–	–	−0.08	0.13	–	–	–	–
*Cyathostomum catinatum*	**−2.11**	**<0**.**01**	**1**.**12**	**<0**.**01**	**−1.29**	**<0**.**01**	**1**.**29**	**0**.**01**	**1**.**15**	**0**.**02**	**−1.46**	**<0**.**01**	0.27	0.52	3.74	0.61	−0.05	0.28	<0.01	0.51	–	–
*Strongylus vulgaris*	**−2.09**	**<0**.**01**	**1**.**50**	**<0**.**01**	**1**.**05**	**0**.**04**	−0.32	0.52	−0.16	0.74	**−2.41**	**<0**.**01**	−0.27	0.57	4.38	0.60	**−0.16**	**< 0.05**	−0.01	0.08	–	–
*Cylicostephanus longibursatus*	**−3.27**	**<0**.**01**	**1**.**57**	**<0**.**01**	**−3.06**	**<0**.**01**	**−2.39**	**<0**.**01**	0.48	0.49	**−1.81**	**0**.**02**	−1.29	0.09	−17.17	0.18	−0.10	0.19	−0.01	0.24	−0.055	0.15
*Cylicocyclus nassatus*^a^	**−1.96**	**<0**.**01**	**1**.**39**	**<0**.**01**	−0.44	0.57	–	–	–	–	**−4.47**	**<0**.**01**	–	–	18.75	0.11	–	–	–	–	–	–
*Coronocyclus coronatus*	**−1.38**	**<0**.**01**	**1**.**21**	**<0**.**01**	**−3.51**	**<0**.**01**	−0.68	0.32	**2**.**59**	**<0**.**01**	−1.05	0.09	−1.01	0.08	–	–	−0.03	0.68	−0.01	0.61	–	–
*Cylicostephanus calicatus*	**−0.91**	**<0**.**01**	**0**.**84**	**<0**.**01**	0.11	0.80	0.29	0.49	0.32	0.45	−0.76	0.06	**−0.88**	**0**.**03**	−11.81	0.11	−0.05	0.29	–	–	–	–
*Cylicocyclus leptostomum*	**−0.81**	**<0**.**01**	**1**.**12**	**<0**.**01**	**−2.52**	**<0**.**01**	**−2.74**	**<0**.**01**	0.85	0.14	**−4.18**	**<0**.**01**	-	-	−14.42	0.23	−0.10	0.11	<0.01	0.88	−0.033	0.33
*Cylicostephanus minutus*	**−0.79**	**<0**.**01**	**1**.**23**	**<0**.**01**	**−5.03**	**<0**.**01**	–	–	**2**.**12**	**<0**.**01**	**−1.17**	**0**.**03**	–	–	–	–	–	–	–	–	–	–
*Cylicocyclus ashworthi*	–	–	**0**.**25**	**0**.**02**	−2.07	0.15	−2.14	0.11	–	–	**−7.68**	**<0**.**01**	**−3.98**	**0**.**01**	24.49	0.32	**−0.38**	**0**.**011**	–	–	−0.14	0.051
*Cylicocyclus insigne*	**0**.**49**	**<0**.**01**	−0.01	0.99	−1.15	0.19	**−1.65**	**0**.**03**	**1**.**74**	**0**.**01**	1.66	0.16	**−5.29**	**<0**.**01**	–	–	−0.05	0.51	**0.03**	**0.01**	−0.050	0.19
*Cylicostephanus goldi*^a^	–	–	**0**.**77**	**<0**.**01**	−0.55	0.37	**−1.22**	**0**.**04**	**2**.**25**	**<0**.**01**	–	–	–	–	–	–	–	–	–	–	–	–
*Coronocyclus labratus*^a^	**0.77**	**<0**.**01**	**1**.**69**	**<0**.**01**	**−1.98**	**0**.**03**	-	-	**13**.**64**	**0**.**02**	**−1.92**	**0**.**02**	–	–	–	–	–	–	**−0.03**	**<0.01**	**−0.11**	**<0.01**
*Coronocyclus labiatus*^a^	−19.39	1.00	**−0.79**	**<0**.**01**	**−9.67**	**<0**.**01**	**−4.69**	**0**.**02**	–	–	3.11	0.19	**−7.58**	**<0**.**01**	–	–	–	–	–	–	**−0.40**	**<0.01**
*Petrovinema poculatum*^a^	−18.62	1.00	**−1.10**	**<0**.**01**	−5.24	0.06	**−6.84**	**0**.**01**	4.30	0.09	–	–	–	–	–	–	–	–	–	–	–	–
*Triodontophorus brevicauda*	−19.28	1.00	**−1.36**	**<0**.**01**	**−25.18**	**<0**.**01**	**8**.**10**	**0**.**03**	**7**.**97**	**0**.**01**	–	–	−1.31	0.55	–	–	0.20	0.42	–	–	−0.16	0.19
*Cylicocyclus elongatus*^a^	−19.74	1.00	**−1.74**	**<0**.**01**	**−26.59**	**<0**.**01**	**10**.**02**	**0**.**04**	**10**.**00**	**<0**.**01**		–	–	–	–	–	–	–	–	–	–	–
*Triodontophorus serratus*	−20.24	1.00	**−2.56**	**<0**.**01**	**−23.52**	**<0**.**01**	**21**.**49**	**<0**.**01**	**18**.**81**	**<0**.**01**	4.52	0.11	−0.95	0.73	–	–	0.33	0.27	0.06	0.50	−0.11	0.46

Significant fixed effects are bolded (*P* < 0.05). Dashes indicate fixed effects that were not present in any top model.

a Indicates species which could not be model averaged due to difficulty in fitting a global model, for which the top models (ΔAIC_c_ = 0) was used for parameter estimates. Dashes indicate fixed effects not present in the top model for these species.

Fecal egg counts decreased from west to east for 9 species (*P* < 0.05, Table 4, Fig. 6). In contrast, no species had higher FEC in the east (Table 4). *Cylicostephanus calicatus*, *Cylicocyclus ashworthi*, *Cylicocyclus insigne* and *Coronocyclus labiatus* were more common in the center of the island (longitude^2^; *P* < 0.05, Table 4).

Compared to horse age and location, other host and environmental factors were not as commonly associated with FEC across parasite species. Sex differences were only observed for *Cyathostomum catinatum* and *Cylicocyclus ashowrthi* with FEC being higher in males than in females (*P* < 0.05, Table 4). FEC of only 2 strongyle species had significant positive associations with local horse density (*Cylicocyclus insigne* and *Coronocyclus labratus*), and only *Coronocyclus labratus* and *Coronocyclus labiatus* decreased in FEC as the field season progressed (Table 4). No significant age:longitude interactions were identified.

### Community analyses

**Figure 7.**
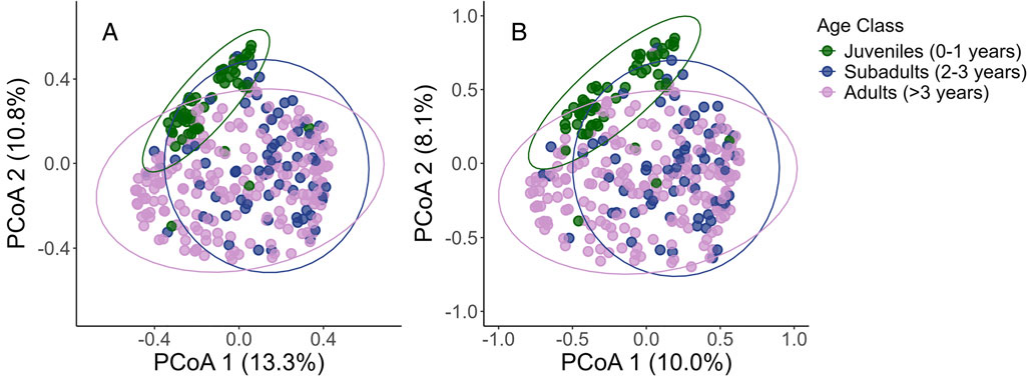
Principal coordinate analysis of the (A) Jaccard and (B) Bray-Curtis dissimilarity matrix of parasitic strongyle community composition among 320 Sable Island horses in 2014. Each point represents a strongyle parasite community, with colour indicating horse age class (Juvenile, Subadult, Adult) with ellipses for the 95% confidence interval for each age class. Discrimination between samples of different ages (in years) are available in the Supplementary Materials.

For the whole population, age explained the greatest proportion of variation in strongyle species composition for both Jaccard (*R*^2^ = 0.11, *P* = 0.001), and Bray–Curtis (*R*^2^ = 0.16, *P* = 0.001) dissimilarities followed by longitude (Jaccard *R*^2^ = 0.03, *P* < 0.01; Bray–Curtis *R*^2^ = 0.041, *P* < 0.01), and sex (Jaccard *R*^2^ = 0.0170, *P* = 0.005; Bray–Curtis *R*^2^ = 0.0191, *P* = 0.002). In contrast, ordinal date and horse density did not explain significant amounts of variation (Table 5). Juveniles (foals and yearlings) and subadults clustered together (age 2–3) appeared to cluster together with age class (Fig. 7), however, adults (age > 3) did not appear to cluster closely (Supplementary Fig. S1).

**Table 5.** Results of permutational analysis of variance (PERMANOVA) testing the influence of host and environmental factors across the whole population and adults (age >3) describing the variation in community composition using Jaccard and Bray-Curtis dissimilarity indexes

	Jaccard dissimilarity index					Bray-curtis dissimilarity index				
	d.f.	SS	*R*^2^	*F*	*P*	d.f.	SS	*R*^2^	*F*	*P*
*Population (n* *=* *320)*										
**poly(Age, 3)**	**3**	**10**.**08**	**0**.**11**	**13**.**00**	**0**.**001**	**3**	**10**.**97**	**0**.**16**	**20**.**84**	**0**.**001**
**poly(Longitude, 2)**	**2**	**2**.**88**	**0**.**030**	**5**.**57**	**0**.**001**	**2**	**2**.**84**	**0**.**041**	**8**.**08**	**0**.**001**
**Sex**	**1**	**0**.**67**	**0**.**007**	**2**.**58**	**0**.**0037**	**1**	**0**.**63**	**0**.**0091**	**3**.**60**	**0**.**0016**
Ordinal date	1	0.26	0.0028	1.02	0.40	1	0.21	0.0030	1.18	0.30
Local horse density	1	0.21	0.0022	0.82	0.65	1	0.10	0.0015	0.59	0.79
Residual	311	80.39	0.85			311	54.58	0.78		
*Adults (n* *=* *193)*										
**poly(Age, 3)**	**3**	**2**.**57**	**0**.**051**	**3**.**60**	**0**.**001**	**3**	**2**.**31**	**0**.**068**	**5**.**00**	**0**.**001**
**poly(Longitude, 2)**	**2**	**2**.**03**	**0**.**040**	**4**.**27**	**0**.**001**	**2**	**1**.**83**	**0**.**054**	**5**.**95**	**0**.**001**
**Social/Reproductive status**	**3**	**1**.**72**	**0**.**034**	**2**.**41**	**0**.**003**	**3**	**1**.**46**	**0**.**043**	**3**.**15**	**0**.**004**
Ordinal date	1	0.14	0.0029	0.60	0.86	1	0.084	0.0025	0.55	0.77
Local horse density	1	0.24	0.0047	1.0012	0.40	1	0.14	0.0042	0.92	0.45
Residual	182	43.28	0.86			182	28.00	0.82		

Significant fixed effects are bolded (*P* < 0.05).

The importance of age and longitude were consistent considering only adults (Table 5), along with social or reproductive status explaining significant, but minimal variation in strongyle community composition (Jaccard *R*^2^ = 0.034, *P* = 0.002; Bray–Curtis *R*^2^ = 0.043, *P* = 0.002). This variation appeared to be driven by females with foals clustering separately from other adults (Supplementary Fig. 2). For adult females models (Supplementary Table S1), age explained the greatest amount of variation in community composition (Jaccard *R*^2^ = 0.074, *P* = 0.001; Bray–Curtis *R*^2^ = 0.093, *P* = 0.0008), followed by longitude (Jaccard *R*^2^ = 0.055, *P* = 0.0016; Bray–Curtis *R*^2^ = 0.068, *P* = 0.0014), and reproductive status (Jaccard *R*^2^ = 0.017, *P* = 0.01; Bray–Curtis *R*^2^ = 0.031, *P* = 0.014). For adult males models (Supplementary Table S11), age explained the most variation (Jaccard *R*^2^ = 0.063, *P* = 0.002; Bray–Curtis *R*^2^ = 0.078, *P* = 0.004), followed by longitude (Jaccard *R*^2^ = 0.049, *P* = 0.001; Bray–Curtis *R*^2^ = 0.063, *P* = 0.001), and local horse density (Jaccard *R*^2^ = 0.018 *P* = 0.020; Bray-Curtis *R*^2^ = 0.022, *P* = 0.023).

Finally, the univariate PERMANOVA only including social band indicated that variation in the composition of strongyle communities were similar within social bands (Jaccard *R*^2^ = 0.37, d.f. = 111, df_residual_ = 208, *P* = 0.004; Bray-Curtis *R*^2^ = 0.38, d.f. = 111, d.f._residual_ = 208, *P* = 0.014).

## Discussion

This study applied a non-invasive method to conduct a comprehensive population-wide study of the drivers of variation in strongyle-parasite communities for a free-living, feral horse population with no individual subjected to anthelmintic treatment. Decomposing aggregate strongyle infections to the species level revealed that host and environmental factors can impact infection patterns differentially across parasite species. Consistent with our prediction, host age was broadly negatively associated with the prevalence and FEC of multiple strongyles. In addition, non-linear patterns of prevalence and FEC were common, highlighting that mixed infections vary across host ontogeny. In contrast, horse sex and life history differences (e.g. social status) appeared to have minimal impact on species-specific infection patterns, nor did they greatly influence the species composition of mixed infections. Horse median location on the island was the only environmental factor that was consistently associated with mixed infections, paralleling a previously reported decrease in aggregate FEC along the west to east gradient of the island (Debeffe *et al*., 2016). Finally, horses in the same social band had similar communities of parasites, emphasizing the possible role of local habitat and space utilization in structuring parasite communities. Taken together, our results reveal that mixed infections are driven by complex associations with host and environmental factors acting uniquely across parasite species.

Our analyses revealed the presence of at least 25 strongyle species infecting Sable Island horses. This is 5 more compared to a previous study that only examined 4 horses (Poissant *et al*., 2021): 4 cyathostomins (*Poteriostomum imparidentatum*, *Cyathostomum pateratum*, *Cylicodontophorus bicoronatus*, *Cylicostephanus bidentatus*) and one strongylin (*Craterostomum acuticaudatum*). As these 5 species have a prevalence of less than 3%, the difference between studies is likely driven by sample coverage. Given that *Cylicocyclus bidentatus* was only found in a single individual, further studies with greater sample coverage across temporal scales may be warranted to verify presence of rare species in this population. Patterns of cyathostomin species prevalence on Sable Island are similar to those of domestic horses (Bellaw and Nielsen, 2020), with high prevalence of core species like *Cyathostomum catinatum, Cylicocyclus nassatus*, *Cylicostephanus longibursatus*, though *Cylicostephanus goldi* is comparatively less common on Sable Island. As noted in Jenkins *et al*. (2020), the high prevalence of *Strongylus* spp. on Sable Island is in contrast to infection patterns of domestic or managed horse populations where these species tend to be rare (Mfitilodze and Hutchinson, 1990; Kuzmina *et al*., 2016; Sargison *et al*., 2022; Abbas *et al*., 2023). One proposed mechanism for the low prevalence of *Strongylus* spp. in managed populations is a dispersal-fecundity trade-off in which high fecundity species (e.g. *Strongylus* spp.; Kuzmina *et al*., 2012) have lower dispersal capacity compared to low-fecundity species (e.g. Cyathostomins) (Sallé *et al*., 2018). Specifically, high fecundity species may be unable to colonize all hosts due to environmental constraints that limit successful dispersal (Sallé *et al*., 2018). However, the high prevalence of *Strongylus* spp. in our study, and other wild horse populations (Harvey *et al*., 2019; Poissant *et al*., 2021), suggest that a dispersal-fecundity trade-off may not be applicable for equine strongyles in the wild. This inference is further supported by the high correlation between mean species-specific FEC and prevalence across strongyle species. Instead, the differences in *Strongylus* spp. prevalence between wild and managed horse populations may be due to anthelmintic use (Saeed *et al*., 2019; Bellaw and Nielsen, 2020). It may be that variation in parasite life history such as prepatent period, migratory behaviour, or encystment patterns may result in differences in susceptibility to anthelmintics. In contrast, other species like *Triodontophorus* spp. were predominantly found in yearlings (consistent with clinical observations; Reinemeyer and Nielsen, 2018), suggesting that certain species-specific infection patterns may be governed by the host (i.e. acquired resistance), as opposed to parasite-specific traits.

Horse age was consistently associated with strongyle infection, with 1–3-year-old horses having the highest parasite prevalence and FEC. Linear and non-linear patterns across horse age were varied across parasite species, contributing to an age-structured pattern of beta diversity. The linear decline in prevalence and FEC across ontogeny indicates increasing strongyle resistance as individuals age, consistent with strongyle infection patterns in domestic (Boisseau *et al*., 2023*a*) and Sable Island (Debeffe *et al*., 2016) horses. Furthermore, significant non-linear age patterns of FEC across multiple species may reflect biological processes that vary across host ontogeny. For example, foals may exhibit lower infection burdens because they have limited parasite exposure (not weaned, thus not consuming as much contaminated herbage), or it may be that certain parasite species have yet to mature due to variation in pre-patent periods among strongyle species (Round, 1969). Once exposed, younger horses may be more susceptible to parasite infections if they have not developed resistance or may have energetic trade-offs between growth and resistance (van der Most *et al*., 2011), resulting in a peak in FEC. As individuals transition to adulthood (age >3), individuals may have had sufficient time to develop resistance, resulting in the decrease in species-specific FEC. Subsequently, adults appear to maintain relatively stable and chronic infections (significant age^3^ associations) for certain species, which may reflect tolerance or the high cost of maintaining resistance (Colditz, 2008).

The cumulation of the various age trends in species-specific FEC resulted in predictable age-structured shifts of parasite community composition, with 1–2-year-olds having the highest species richness, followed by a decrease in older individuals. This is consistent with classic succession theory in which communities progress to a climax state followed by a decrease in complexity to a stable plateau (Miller and TerHorst, 2012). Succession patterns have been observed in microparasite communities of other long-lived mammals (African buffalo; *Syncerus caffer*) (Glidden *et al*., 2023) and could be a consequence of temporally mediated niche differentiation (Dini-Andreote *et al*., 2014). Similar changes in the composition of parasite communities across host age were also documented in other wild systems such as helminth parasites of Soay Sheep (Craig *et al*., 2006) and helminth and coccidian parasites of wild zebra and springbok (Turner and Getz, 2010). Such shifts highlight the dynamic nature of mixed infections which can be obscured when using aggregate measures of infections. For instance, *Strongylus* spp. is likely to disproportionately influence trends in aggregate strongyle FEC in Sable Island horses, masking species-specific patterns of less abundant species. Aggregate FEC would not be able to detect that *Cyathostomum catinatum* FEC is stable across ontogeny, or that *Triodontophorus* spp. FEC peak in yearlings. Species-specific patterns across host age may provide critical context to assess the health of animals, for example, while the presence of *Triodontophorus* spp. is typical in 1–2 year olds, its presence in adults could indicate weakened immunity.

Prevalence and FEC for most parasite species were higher on the western side of the island, broadly consistent with previously reported patterns observed for strongyle FEC in this population (Debeffe *et al*., 2016; Gold *et al*., 2019). Proposed explanations for this west to east decline include greater water availability and vegetation density on the western side of the island (Contasti *et al*., 2012; Rozen-Rechels *et al*., 2015) which may enhance the viability of parasite larvae in the environment (Debeffe *et al*., 2016). These patterns may also be explained by the heterogeneous distribution of horses along the island. In particular, overlapping home ranges are known to influence parasite species sharing among wild ungulate species (Stephens *et al*., 2019). Thus, non-linear prevalence patterns may be driven by horses in the center of the island having more overlapping home ranges with horses from both the east and the west of the island. Host space utilization may also explain certain opposing trends in parasite abundance across the island. There were a handful of species that increased in prevalence towards the east, opposite of the patterns observed in overall strongyle burden (Debeffe *et al*., 2016), which may be a consequence of variation in host population demography. For example, *Triodontophorus serratus* is predominantly found in yearlings, and there is a greater density of yearlings in the eastern half (Contasti *et al*., 2013).

Of all variables considered, social band explained the greatest proportion of among-individual variation in the composition of strongyle communities. For environmentally transmitted parasites such as strongyles, social groups may be exposed to the same parasites since they share the same habitat. Such patterns have been observed in the gastrointestinal parasite communities of meerkats (*Suricata suricatta*; Leclaire and Faulkner, 2014), and are broadly consistent with expectations that shared habitat utilization (i.e. sympatry) result in similar parasite community between host species (Archie and Ezenwa, 2011; Tombak *et al*., 2021; Beaumelle *et al*., 2022). A similar influence of social group was observed for gastrointestinal microbiome communities of Sable Island horses (Stothart *et al*., 2021). Though bacteria can be directly transmitted between individuals (e.g. *via* social grooming), this is not the case for strongylid nematodes, and it is likely that the similar strongyle parasite communities is due to grazing in shared habitats that are contaminated by strongyle larvae. Given that the gastrointestinal microbiome of horses can modulate the host response to strongyle infection (Boisseau *et al*., 2023*b*), it may also be the case that social bands effects are being modulated by microbiome diversity. Finally, since FEC is heritable in Sable Island horses (Gold *et al*., 2019), social band effects could also be partly due to the fact that bands often contain relatives (parent-offspring pairs and siblings).

Species-specific differences in seasonal trends have been documented for equine strongyles (Sargison *et al*., 2022; Abbas *et al*., 2023), but only 4 species in our study exhibited significant variation across the field season. This, in turn, translated to no significant shifts in the composition of parasite communities across ordinal date for either Jaccard and Bray–Curtis dissimilarities. Our contrasting result likely reflects the small sampling window of this study (July 23–September 5). Additionally, it should be noted that our results are based on samples from a single year. Longitudinal variation in infection can differ by parasite species (e.g. strongyles *vs* coccidia in Soay Sheep; Hayward *et al*., 2022) and parasite community composition can change across years (e.g. parasite communities of minnows; Hirtle *et al*., 2023). Though long-term changes in non-strongylid parasite species of domestic horses are recognized (Tolliver *et al*., 1987; Sallé *et al*., 2020), there is limited species-specific information for most strongyle species. Though aggregate strongyle FEC does not appear to vary much over consecutive years for Sable Island horses (Gold *et al*., 2019), inter-annual variation in species-specific FEC have yet to be tested. Longer-term studies will be necessary to test if the observed stability of strongyle FEC in Sable Island horses conceal long-term species-specific patterns.

Our results indicated limited systematic differences in species-specific prevalence and FEC between the sexes, as in domestic equines (Sallé *et al*., 2018). Although sex differences in parasite infection may be expected due to physiological differences (e.g. testosterone acting as an immune suppressor (Ezenwa *et al*., 2012)), differences between sexes in Sable Island horses appears to be limited to variation in life history, with females with foals having different parasite communities from all other adults (Supplementary Fig. S2). Such patterns are consistent with aggregate strongyle FECs of Sable Island horses (Debeffe *et al*., 2016), and suggest that sex-specific variation in life history may drive parasite community composition. However, the amount of variation explained by reproductive or social status were minimal relative to other factors such as age and location. Similar results were seen for local host density, with significant, but minimal, variation in species composition attributed to density for adult males, paralleling patterns of aggregate FEC seen in Sable Island horses (Debeffe *et al*., 2016).

While DNA metabarcoding has clear benefits for the study of mixed infections, it also has limitations. For example, index hopping can lead to false positives (Wright and Vetsigian, 2016), and the sequencing of a limited number of larvae may prevent the detection of low abundance species. Index hopping could be mitigated in future studies by using dual index primers (Wright and Vetsigian, 2016). Furthermore, detection of individual species could possibly be improved using eDNA approaches using feces, as metabarcoding using DNA extracted from feces is possibly sensitive enough to identify the presence of parasite species even in the absence of eggs or larvae (Davey *et al*., 2023).

Another common limitation of DNA metabarcoding studies is the inability to account for species-level variation in gene copy numbers, PCR efficiency and number of cells on abundance estimates (Avramenko *et al*., 2015; Poissant *et al*., 2021). To account for this, a novel approach was implemented where correction factors of multiple species were estimated concurrently in a single regression model, as opposed to multiple species-specific regressions. This approach suggested that the relative proportion of *Strongylus vulgaris* was heavily overestimated when using molecular characterization, with a correction factor of 4.66. The other *Strongylus* spp. were also overestimated with molecular characterization, but to a lesser extent. The correction factors calculated in this study are higher in magnitude compared to a previous study with less samples (Poissant *et al*., 2021), but are consistent in relative biases between species. As noted previously (Poissant *et al*., 2021), the overestimation of *Strongylus* spp. using molecular methods are likely a reflection of variation in cell number among species since *Strongylus* spp. larvae have a greater number of cells, especially *S. vulgaris* which has 1.5–4 times more cells than other species. The inability to identify cyathostomins larvae to species using morphology prevented us from calculating species-specific correction factors for this group in the current study. However, a possible solution to this would be to identify individual larvae using DNA barcoding, and subsequently assessing their relative ITS-2 amplification efficiency using qPCR (Courtot *et al*., 2023) or by generating mock communities (Avramenko *et al*., 2015).

This study highlights that the prevalence and egg counts of parasite species respond uniquely across host and environmental factors in feral horses. Variation in mixed strongyle infections was shown to vary across host age, with shifts occurring in younger individuals followed by relative stability in adulthood. Prevalence and FEC of some species also varied across the length of Sable Island, emphasizing the role of host habitat use on parasite infections. Furthermore, species-specific data, as reported here, suggest that aggregate measures of parasite infection can be dominated by species with high fecundity and prevalence, which masks species-specific infection patterns of other species. As different parasite species can result in different pathologies and alter epidemiological patterns, knowledge of variation in species-specific infection among individuals will provide valuable context for quantifying the consequences of parasites in host populations.

## Supporting information

Ahn et al. supplementary material Ahn et al. supplementary material

## Data Availability

Nemabiome DNA metabarcoding data have been deposited in the NCBI SRA under the BioProject accession code PRJNA1190982. Detailed sample metadata and code will be available from the corresponding authors upon reasonable request.

## Supplementary material

The supplementary material for this article can be found at https://doi.org/10.1017/S0031182024001185.
