# Supplementary material for: Mixed strongyle parasite infections vary across host age and space in a population of feral horses
Source: Parasitology. 2024 Dec 12;151(12):1299–316. doi: 10.1017/S0031182024001185 (PMC11894017; doi:10.1017/S0031182024001185)
Supplement: Ahn et al. supplementary material [file S0031182024001185sup001.docx]

**Supplementary Results**

**Supplementary Table S1.** Results of sex-specific permutational analysis of variance (PERMANOVA) testing the influence of host and environmental factors across of adult (age > 3) in describing the variation in community composition using Jaccard and Bray-Curtis dissimilarity indexes. Significant fixed effects are bolded (*P* < 0.05)

|  | Jaccard Dissimilarity Index | | | | |  | Bray-Curtis Dissimilarity Index | | | | |
| --- | --- | --- | --- | --- | --- | --- | --- | --- | --- | --- | --- |
|  | df | SS | R^2^ | *F* | *p* |  | df | SS | R^2^ | *F* | *p* |
|  |  |  |  |  |  |  |  |  |  |  |  |
| *Adult Females (n = 82)* |  |  |  |  |  |  |  |  |  |  |  |
| **poly(Age, 3)** | **3** | **1.64** | **0.074** | **2.19** | **0.010** |  | **3** | **1.43** | **0.093** | **2.90** | **0.0008** |
| **poly(Longitude,2)** | **2** | **1.22** | **0.055** | **2.44** | **0.0016** |  | **2** | **1.04** | **0.068** | **3.18** | **0.0014** |
| **Reproductive Status** | **1** | **0.58** | **0.026** | **2.31** | **0.017** |  | **1** | **0.48** | **0.031** | **2.93** | **0.014** |
| Ordinal Date | 1 | 0.095 | 0.0043 | 0.38 | 0.99 |  | 1 | 0.044 | 0.0029 | 0.27 | 0.96 |
| Local Horse Density | 1 | 0.015 | 0.0015 | 1.35 | 0.18 |  | 1 | 0.28 | 0.018 | 1.69 | 0.13 |
| Residual | 73 | 18.22 | 0.82 |  |  |  | 73 | 12.00 | 0.78 |  |  |
|  |  |  |  |  |  |  |  |  |  |  |  |
| *Adult Males (n = 111)* |  |  |  |  |  |  |  |  |  |  |  |
| **poly(Age, 3)** | **3** | **1.70** | **0.063** | **2.50** | **0.002** |  | **3** | **1.40** | **0.078** | **3.23** | **0.004** |
| **poly(Longitude,2)** | **2** | **1.32** | **0.049** | **2.90** | **0.001** |  | **2** | **1.13** | **0.063** | **3.90** | **0.001** |
| Social Status | 1 | 0.29 | 0.011 | 1.28 | 0.20 |  | 1 | 0.20 | 0.011 | 1.42 | 0.19 |
| Ordinal Date | 1 | 0.18 | 0.0068 | 0.81 | 0.61 |  | 1 | 0.12 | 0.0065 | 0.81 | 0.55 |
| **Local Horse Density** | **1** | **0.50** | **0.018** | **2.19** | **0.020** |  | **1** | **0.39** | **0.022** | **2.72** | **0.023** |
| Residual | 102 | 23.19 | 0.85 |  |  |  | 102 | 14.71 | 0.82 |  |  |


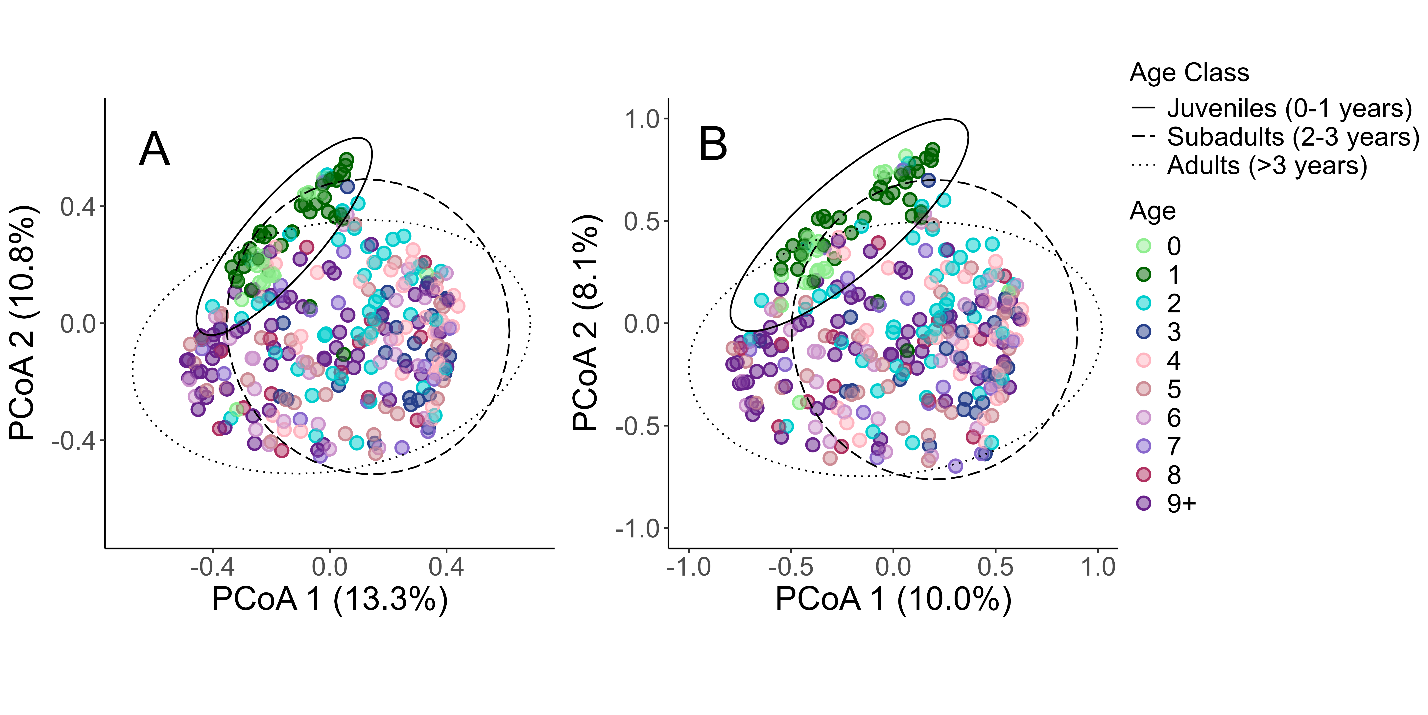
**Supplementary Figure S1.** Principal coordinate analysis of the (A) Jaccard and (B) Bray-Curtis dissimilarity matrix of parasitic strongyle community composition among 320 Sable Island horses in 2014. Each point represents a strongyle parasite community, with colour indicating horse age (in years) with ellipses for the 95% confidence interval for each age class (Juvenile, Subadult, Adult).


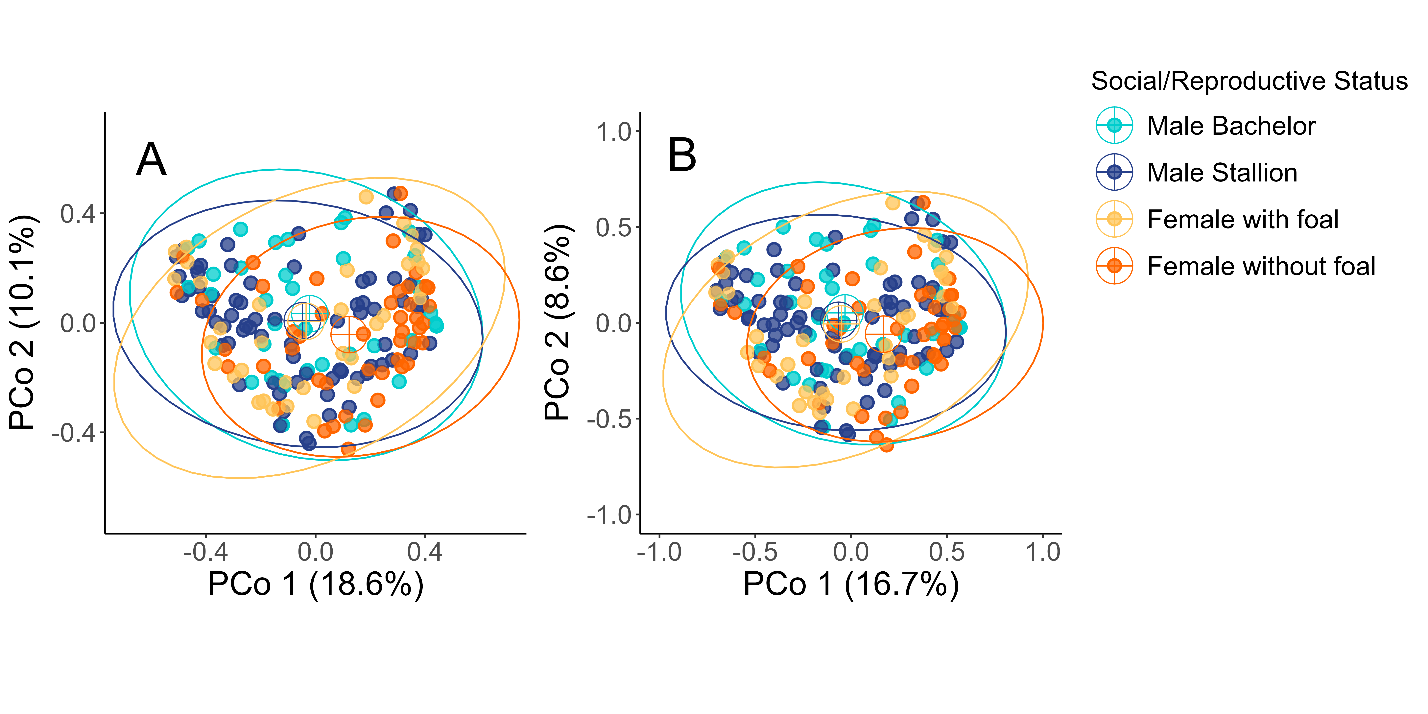
**Supplementary Figure S2.** Principal coordinate analysis of the (A) Jaccard and (B) Bray-Curtis dissimilarity matrix of parasitic strongyle community composition among 193 adult Sable Island horses (age > 3) in 2014. Each point represents a strongyle parasite community, with colour indicating the male social status (bachelor or stallion) or female reproductive status (with or without foal). Group means are indicated by cross-hairs.
